# Comparative evaluation of machine learning models for enhancing diagnostic accuracy of otitis media with effusion in children with adenoid hypertrophy

**DOI:** 10.3389/fped.2025.1614495

**Published:** 2025-06-19

**Authors:** Xiaote Zhang, Qiaoyi Xie, Ganggang Wu

**Affiliations:** ^1^ Department of Otolaryngology Head and Neck Surgery, Ningbo Yinzhou No.2 Hospital, Ningbo, Zhejiang, China; ^2^ Department of Pediatrics, The Affiliated People’s Hospital of Ningbo University, Ningbo, Zhejiang, China

**Keywords:** otitis media with effusion, adenoid hypertrophy, machine learning, random forest, diagnostic accuracy, SHAP analysis

## Abstract

**Background:**

Otitis media with effusion (OME) affects a significant proportion of children with adenoid hypertrophy (AH) and can lead to developmental sequelae when chronic. Current non-invasive screening modalities rely predominantly on acoustic immittance measurements, which demonstrate variable diagnostic performance. Given the urgent need for improved diagnostic methods and extensive characterization of risk factors for OME in AH children, developing diagnostic models represents an efficient strategy to enhance clinical identification accuracy in practice.

**Objective:**

This study aims to develop and validate an optimal machine learning (ML)-based prediction model for OME in AH children by comparing multiple algorithmic approaches, integrating clinical indicators with acoustic measurements into a widely applicable diagnostic tool.

**Methods:**

A retrospective analysis was conducted on 847 pediatric patients with AH. Five ML algorithms were developed to identify OME using demographic, clinical, laboratory, and acoustic immittance parameters. The dataset underwent 7:3 stratified partitioning for training and testing cohorts. Within the training cohort, models were initially optimized through randomized grid search with 5-fold cross-validation, followed by comprehensive training with optimized parameters. Model performance was evaluated in the testing cohort using discrimination, calibration, clinical utility metrics, and confusion matrix-derived statistics. The optimal ML model was subsequently analyzed through SHapley Additive exPlanations (SHAP) methodology for interpretability, with sequential ablation testing performed to identify critical predictive variables.

**Results:**

Among 847 children with AH, 262 (30.9%) were diagnosed with OME. The Random Forest (RF) model demonstrated superior performance with the highest discrimination (area under the receiver operating characteristic curve = 0.919), balanced calibration (Brier score = 0.102), and optimal clinical utility across decision thresholds of 0.4–0.6. Confusion matrix analysis further confirmed RF as the optimal model, achieving 0.875 accuracy and robust inter-rater agreement (Cohen's kappa coefficient = 0.696) in the testing cohort. SHAP analysis identified the adenoid-to-nasopharyngeal ratio as the predominant diagnostic indicator, followed by tympanometric type and history of recurrent respiratory infections.

**Conclusion:**

An RF-based diagnostic model effectively identifies OME in AH children by integrating anatomical, functional, and inflammatory parameters, providing a clinically applicable tool for enhanced diagnostic accuracy and evidence-based management decisions.

## Introduction

Otitis media with effusion (OME) represents one of the most common childhood conditions, with approximately 2.2 million new cases diagnosed annually in the United States (1). While most episodes resolve spontaneously within 3 months, about 25% of cases persist for ≥3 months, classified as chronic OME (2). It represents the leading cause of acquired hearing loss in pediatric populations and may be associated with significant developmental sequelae including speech delays, vestibular disturbances, behavioral problems, and educational difficulties (3, 4). The etiology of OME is multifactorial, with adenoid hypertrophy (AH) established as a primary contributor in young children (5). AH mechanically obstructs the Eustachian tube, creating negative middle ear pressure, and serves as a pathogen reservoir, facilitating the retrograde migration of microorganisms into the middle ear, disrupting mucosal function, and promoting persistent effusion (6).

Despite the established relationship between AH and OME, accurate identification of OME in AH children presents significant challenges. Young children demonstrate limited ability to recognize and articulate subtle hearing changes, complicating early detection by caregivers. While tympanocentesis represents the diagnostic gold standard, its invasive nature precludes widespread implementation for screening purposes (7). Among non-invasive alternatives, acoustic immittance measurement has gained prominence due to its simplicity, brief administration time, minimal cooperation requirements, and widespread availability across healthcare settings (8). However, conventional tympanometry demonstrates important limitations in diagnostic reliability, with area under the receiver operating characteristic curve (AUROC) values ranging from 0.68 to 0.93 in detecting middle ear effusion (9–12). These diagnostic uncertainties highlight the need for more accurate, child-appropriate diagnostic methods to identify AH children at risk for developing OME.

While wideband acoustic immittance represents a promising advancement in tympanometric assessment, its clinical integration faces significant temporal constraints (13, 14). These technologies remain in early validation phases, with widespread implementation delayed by requirements for additional efficacy studies, specialized equipment procurement, and healthcare provider training. This implementation timeline creates an urgent diagnostic gap for the substantial population of AH children who require immediate, accurate assessment for OME during critical developmental windows. In contrast, extensive research has thoroughly characterized risk factors for OME development in AH children, providing a robust knowledge foundation (15–17); yet this evidence remains underutilized in clinical practice due to the absence of validated predictive instruments. With the rapid advancement of artificial intelligence, particularly machine learning (ML) technologies, a timely solution emerges to bridge this implementation gap (18, 19). ML algorithms can rapidly process multidimensional clinical data, identifying complex patterns and relationships between variables that conventional statistical approaches may fail to capture (20–24). These computational techniques enable efficient integration of readily available clinical indicators into unified predictive frameworks that can be rapidly deployed across all levels of healthcare facilities.

Therefore, this study aims to develop and validate an optimal ML-based diagnostic model for OME in AH children by comparing the performance of multiple algorithmic approaches. By incorporating readily available clinical indicators with acoustic immittance findings, we seek to create a practical, non-invasive diagnostic tool implementable across various healthcare settings. The resulting model will facilitate individualized risk stratification, guiding appropriate clinical interventions while minimizing unnecessary invasive procedures, ultimately supporting evidence-based management decisions in this vulnerable pediatric population.

## Materials and methods

### Study design

The study protocol received formal ethical approval from the Ethics Committee of Ningbo Yinzhou No.2 Hospital (2025-014) and adhered to all principles established in the Declaration of Helsinki. Given the retrospective, observational design, the ethics committee granted a waiver of individual informed consent. Patient confidentiality was maintained through comprehensive deidentification procedures, with systematic removal of all personal identifiers from electronic health records prior to analysis in accordance with institutional privacy standards.

Sample size determination followed established methodological principles for predictive model development. Based on previous studies and clinical experience (25), we estimated the prevalence of OME among AH children at approximately 30%. Adhering to the recommended minimum of 10 events per predictor variable to minimize overfitting risk, and planning to evaluate up to 10 potential predictors (26), we calculated a required minimum cohort of 333 patients. This sample size would yield approximately 100 cases with confirmed OME, ensuring sufficient statistical power for robust model development and internal validation.

### Study population

Consecutive pediatric patients diagnosed with AH at our institution were enrolled from January 2021 until 1,000 cases were identified in January 2025. From this cohort, patients were included in the study if they: (1) were aged 3–12 years; (2) had complete clinical documentation; and (3) received confirmed AH diagnosis. Exclusion criteria encompassed: (1) alternative causes of hearing abnormalities, including cranial trauma, middle or inner ear injury, or congenital hearing impairment; (2) craniofacial anomalies attributable to other conditions such as Down syndrome or congenital cleft palate; or (3) severe underlying systemic disease. Bilateral acoustic immittance testing and lateral nasopharyngeal radiography were performed on all subjects. AH was diagnosed when the adenoid-to-nasopharyngeal (A/N) ratio measured from lateral radiographs exceeded 0.70 (27–29), with this parameter also utilized to quantify hypertrophy severity. OME diagnosis was established through a two-step protocol involving bilateral acoustic immittance measurement followed by otoendoscopic examination when indicated. Tympanograms were categorized as normal (bilateral Type A) or abnormal (Type B or C) (30). Patients exhibiting abnormal tympanometric findings underwent confirmatory otoendoscopic examination to exclude cerumen impaction and confirm middle ear effusion; those with confirmed effusion were classified as the AH + OME Group, while subjects with normal tympanograms or those with abnormal tympanograms but no effusion on otoendoscopy were designated as the AH Group.

### Potential predictors

Multiple potential predictive variables were systematically extracted from electronic medical records. Demographic and clinical history data included age, gender, duration of AH-related symptoms (including nasal obstruction, mouth breathing, and snoring), and body mass index (BMI). Physical examination findings incorporated tonsil size grading. Environmental and comorbidity factors were documented, including passive smoke exposure, chronic rhinosinusitis, allergic rhinitis, asthma, and history of recurrent respiratory infections (defined as ≥6 episodes within 12 months). Laboratory parameters included comprehensive hematologic assessment comprising differential leukocyte distribution (neutrophil, lymphocyte, monocyte, eosinophil, and basophil percentages) and quantitative Total IgE measurements.

### Data preprocessing and partitioning

The dataset underwent stratified partitioning to maintain proportional representation of OME cases, with 70% allocated to model training and 30% reserved for independent testing. This stratification process preserved the outcome distribution across subsets while ensuring statistical independence between cohorts. Balanced distribution of patient characteristics between partitions was confirmed using standardized mean differences, with values below 0.1 considered indicative of adequate equilibrium. Feature preprocessing employed an adaptive standardization protocol based on distribution characteristics. Variables exhibiting approximately normal distributions (skewness < 2, kurtosis < 7) underwent *z*-score normalization to center at zero with unit standard deviation. For non-normally distributed variables or those with substantial outliers (>10%), robust scaling was implemented to achieve median centering with interquartile range normalization, thereby minimizing the influence of extreme values while preserving relative relationships. Categorical variables received targeted encoding: binary factors were processed through dichotomous encoding (0/1), while ordinal variables underwent sequential encoding to preserve their inherent hierarchical relationships.

### Model training and hyperparameter tuning

Five ML algorithms were implemented for OME identification in AH children. Logistic regression (LR) was selected for its transparent coefficient interpretation and established clinical utility; random forest (RF) and eXtreme Gradient Boosting (XGBoost) were incorporated for their ability to model complex non-linear relationships and quantify variable importance; support vector machine (SVM) was included for its efficacy in handling high-dimensional feature spaces; and K-nearest neighbors (KNN) was employed to capture local patterns through its instance-based learning approach. Algorithm-specific hyperparameter optimization was executed through randomized grid search with 5-fold cross-validation. The parameter space for LR encompassed regularization strength and penalty type; RF optimization targeted maximum tree depth, estimator count, and minimum samples per leaf; XGBoost tuning addressed learning rate, maximum depth, and estimator quantity; SVM optimization included kernel selection, regularization parameter, and kernel coefficient; and KNN parameter tuning focused on neighbor count, distance metric, and weighting function. Each algorithm underwent 100 iterations of systematic parameter exploration using validation loss as the optimization metric to ensure optimal model configuration. Final models were then trained on the complete training dataset with these optimized parameters to maximize statistical power and enhance generalizability.

### Model performance evaluation

Comparative assessment of algorithm performance was executed in the independent testing cohort using a multi-faceted evaluation framework. Discriminative capacity was quantified through AUROC analysis. Calibration curves and Brier scores (BS) were employed to assess the correspondence between predicted probabilities and observed outcomes, quantifying the models' potential over- or under-estimation tendencies. Clinical utility was examined through decision curve analysis (DCA), which assessed net benefit across a range of clinically relevant threshold probabilities while accounting for intervention consequences. Threshold-dependent performance was characterized using sensitivity, specificity, positive predictive value (PPV), negative predictive value (NPV), and overall accuracy at clinically determined decision thresholds. Additionally, Cohen's Kappa coefficients were calculated to measure inter-rater reliability between predicted and observed outcomes, providing a metric of agreement that accounts for chance concordance in classification tasks. Based on this comprehensive evaluation protocol, the algorithm demonstrating optimal performance across multiple metrics was identified as the preferred prediction model for clinical implementation.

### Interpretability analysis

The mechanistic underpinnings of the optimal prediction model were elucidated through comprehensive interpretability analysis employing SHapley Additive exPlanations (SHAP) methodology. SHAP values were calculated to quantify individual feature contributions based on cooperative game theory principles (31). Global feature importance was determined by computing mean absolute SHAP values across the testing cohort, identifying the relative contribution of each predictor to model discrimination. SHAP summary bar plots were generated to visualize the relative importance of features, while SHAP beeswarm plots were constructed to illustrate both magnitude and directionality of feature effects, characterizing their influence on OME probability. To validate these findings, sequential feature ablation testing was conducted by iteratively removing important variables and quantifying performance changes through confusion matrix metrics, thereby confirming the practical significance of identified predictors.

### Statistical analysis

Statistical evaluations were conducted using methodologies aligned with variable distribution characteristics. Descriptive statistics were presented as frequencies with percentages for categorical variables and as medians with interquartile ranges for continuous variables given their predominantly non-normal distributions. Between-group comparisons employed chi-square or Fisher exact tests for categorical variables and Mann–Whitney *U* tests for continuous measures. Statistical significance was established at *p* < 0.05. All analyses were implemented in Python (version 3.12.0) utilizing scikit-learn (version 1.4.0) for machine learning operations and matplotlib (version 3.8.0) for visualization.

## Results

### Patient characteristics

**Figure 1:**
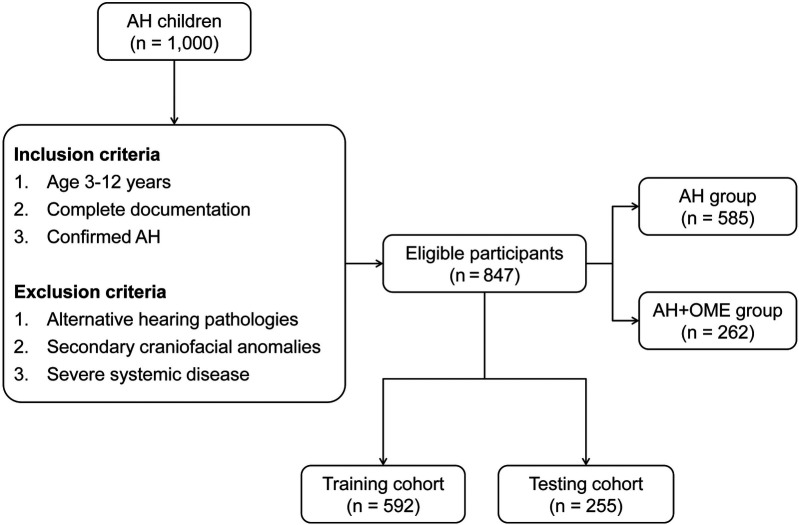
Patient recruitment and study flow diagram.

Among the 1,000 AH children initially evaluated, 847 met eligibility criteria after applying inclusion and exclusion parameters (Figure 1). Within this cohort, 262 patients (30.9%) were diagnosed with OME comorbid with AH, while 585 children presented with isolated AH. Comparative analysis of clinical characteristics between these groups revealed several significant predictors associated with OME development, as detailed in Table 1. Children in the AH + OME group exhibited higher BMI and longer symptom duration compared to the AH group. Advanced tonsillar hypertrophy was more prevalent among OME patients, with Grade 3 tonsils being notably overrepresented. Comorbid conditions were observed with significantly greater frequency in the AH + OME group, including allergic rhinitis, asthma, chronic rhinosinusitis, passive smoke exposure, and recurrent respiratory infections. Laboratory parameters revealed elevated monocyte and neutrophil percentages, along with increased total IgE levels in the AH + OME group. The A/N ratio was significantly higher in AH + OME patients. Tympanometric findings differed markedly between groups, with Type B tympanograms predominating in the AH + OME group.

**Table 1:** Comparison of clinical characteristics between AH group and AH + OME group.

Variable		AH group (*n* = 585)	AH + OME group (*n* = 262)	Statistic	*p*-value
Age, years		5 (4, 8)	4 (4, 6)	0.252^a^	0.795
BMI, kg/m^2^		16.1 (12.3, 17.9)	17.2 (15.2, 18.9)	2.332^a^	0.015
Duration of AH-related symptoms, months		12 (5, 30)	15 (6, 25)	6.021^a^	0.003
Gender, *n* (%)	Female	278 (47.5%)	114 (43.5%)	1.170^b^	0.279
	Male	307 (52.5%)	148 (56.5%)		
Tonsil size, *n* (%)	Grade 0	45 (7.7%)	23 (8.8%)	19.590^b^	0.001
	Grade 1	126 (21.5%)	37 (14.1%)		
	Grade 2	241 (41.2%)	87 (33.2%)		
	Grade 3	165 (28.2%)	111 (42.4%)		
	Grade 4	8 (1.4%)	4 (1.5%)		
Allergic rhinitis, *n* (%)	Yes	393 (67.2%)	198 (75.6%)	6.044^b^	0.014
	No	192 (32.8%)	64 (24.4%)		
Asthma, *n* (%)	Yes	98 (16.8%)	67 (25.6%)	8.975^b^	0.003
	No	487 (83.2%)	195 (74.4%)		
Chronic rhinosinusitis, *n* (%)	Yes	122 (20.9%)	82 (31.3%)	10.793^b^	0.001
	No	463 (79.1%)	180 (68.7%)		
Passive smoke exposure, *n* (%)	Yes	155 (26.5%)	101 (38.6%)	12.467^b^	<0.001
	No	430 (73.5%)	161 (61.4%)		
Recurrent respiratory infections, *n* (%)	Yes	389 (66.5%)	196 (74.8%)	5.854^b^	0.016
	No	196 (33.5%)	66 (25.2%)		
Basophil, %		0.5 (0.2, 0.7)	0.5 (0.3, 0.8)	1.167^a^	0.219
Eosinophil, %		3.1 (2.0, 5.7)	3.0 (2.1, 5.6)	0.083^a^	0.934
Lymphocyte, %		45.4 (30.4, 55.5)	44.0 (29.4, 54.2)	1.228^a^	0.130
Monocyte, %		4.9 (3.9, 5.9)	5.2 (4.1, 6.2)	2.477^a^	0.013
Neutrophil, %		44.4 (35.6, 55.3)	46.8 (36.1, 56.8)	2.051^a^	0.040
Total IgE, IU/ml		55.5 (30.5, 96.1)	68.1 (37.6, 108.4)	2.672^a^	0.008
A/N ratio		0.79 (0.72, 0.87)	0.84 (0.76, 0.92)	17.033^a^	<0.001
Tympanometric type, *n* (%)	Type A	443 (75.7%)	31 (11.8%)	305.059^b^	<0.001
	Type B	76 (13.0%)	150 (57.3%)		
	Type C	66 (11.3%)	81 (30.9%)		

Continuous variables are presented as median (interquartile range). AH, adenoid hypertrophy; OME, otitis media with effusion; BMI, body mass index; IgE, immunoglobulin E; A/N ratio, adenoid-to-nasopharyngeal ratio; Type A, normal tympanogram; Type B, flat tympanogram; Type C, negative pressure tympanogram.

^a^ For Mann–Whitney *U* test.

^b^ For chi-square test.

For model training and validation purposes, the study population was stratified into training (*n* = 592) and testing (*n* = 255) cohorts, with 183 (30.9%) and 79 (30.9%) OME cases distributed proportionally between groups. No statistically significant differences were observed between the training and testing cohorts across all evaluated parameters (all *p* > 0.05), as detailed in Supplementary Table S1.

### Model training and optimization

Five distinct ML algorithms were implemented to identify OME in AH patients: LR, RF, XGBoost, SVM, and KNN. The optimization process was visualized in Supplementary Figure S1, with training and validation loss trajectories indicating appropriate model fitting and no significant overfitting across all algorithms. The comprehensive hyperparameter configurations determined through systematic cross-validation are detailed in Supplementary Table S2. These optimized parameters were subsequently employed for final model training on the complete training dataset.

### Model performance comparison

**Figure 2:**
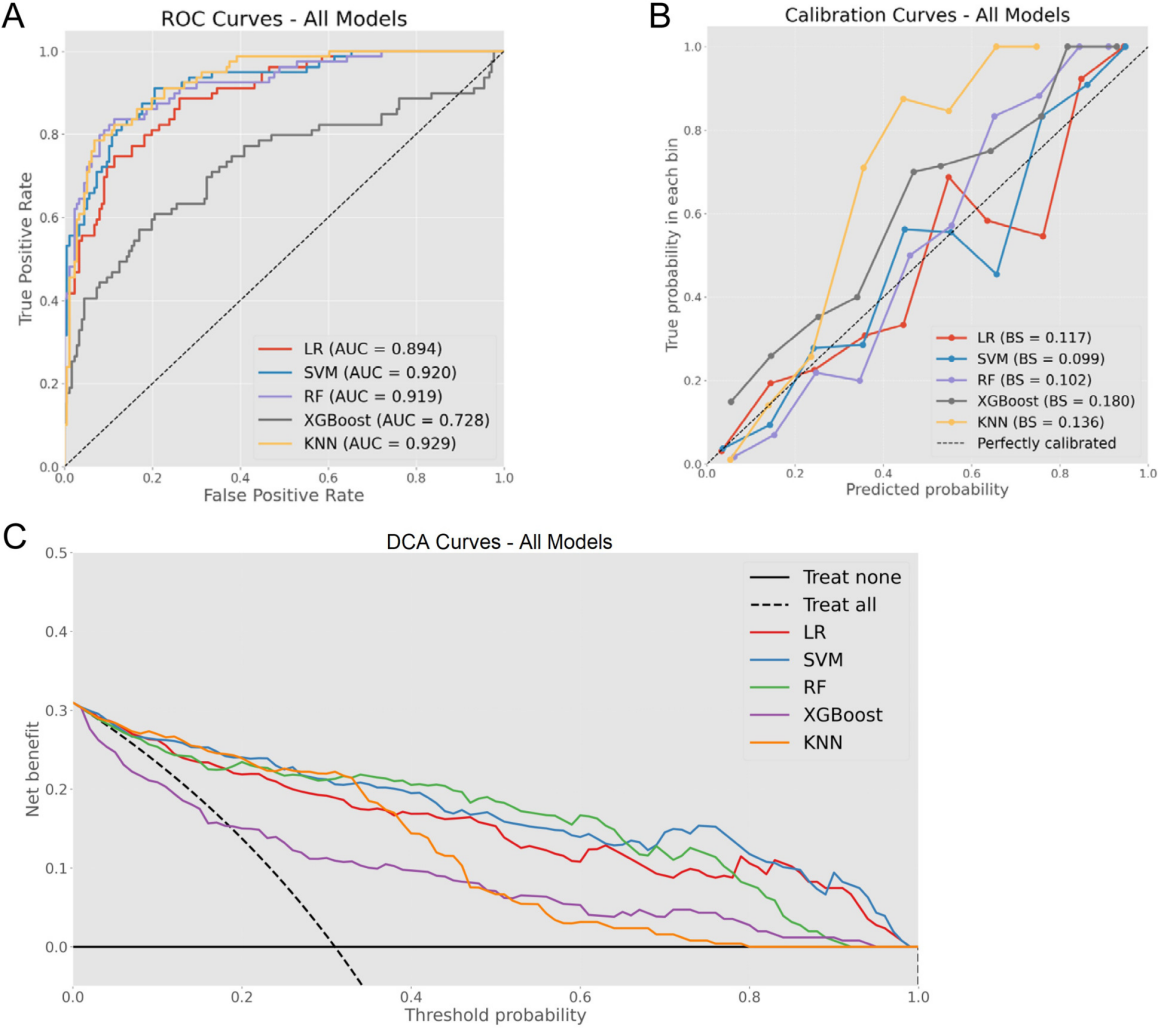
Comprehensive model evaluation metrics. **(A)** ROC curves for all models with corresponding AUC values. **(B)** Calibration curves demonstrating the relationship between predicted and observed probabilities across models. **(C)** DCA illustrating clinical net benefit across various threshold probabilities.

A comprehensive evaluation of the five ML models revealed varying capabilities in OME risk stratification, as shown in Figure 2, which illustrates model performance across three critical dimensions: discrimination ability, calibration accuracy, and clinical utility. In the testing cohort, ROC curve assessment identified SVM, RF, and KNN as superior performers with AUROC values exceeding 0.9. Calibration curve analysis subsequently eliminated KNN due to substantial probability underestimation, while RF and SVM maintained balanced calibration profiles. DCA further differentiated between the remaining contenders, with RF demonstrating superior net benefit across the clinically relevant threshold probability range (0.4–0.6).

**Figure 3:**
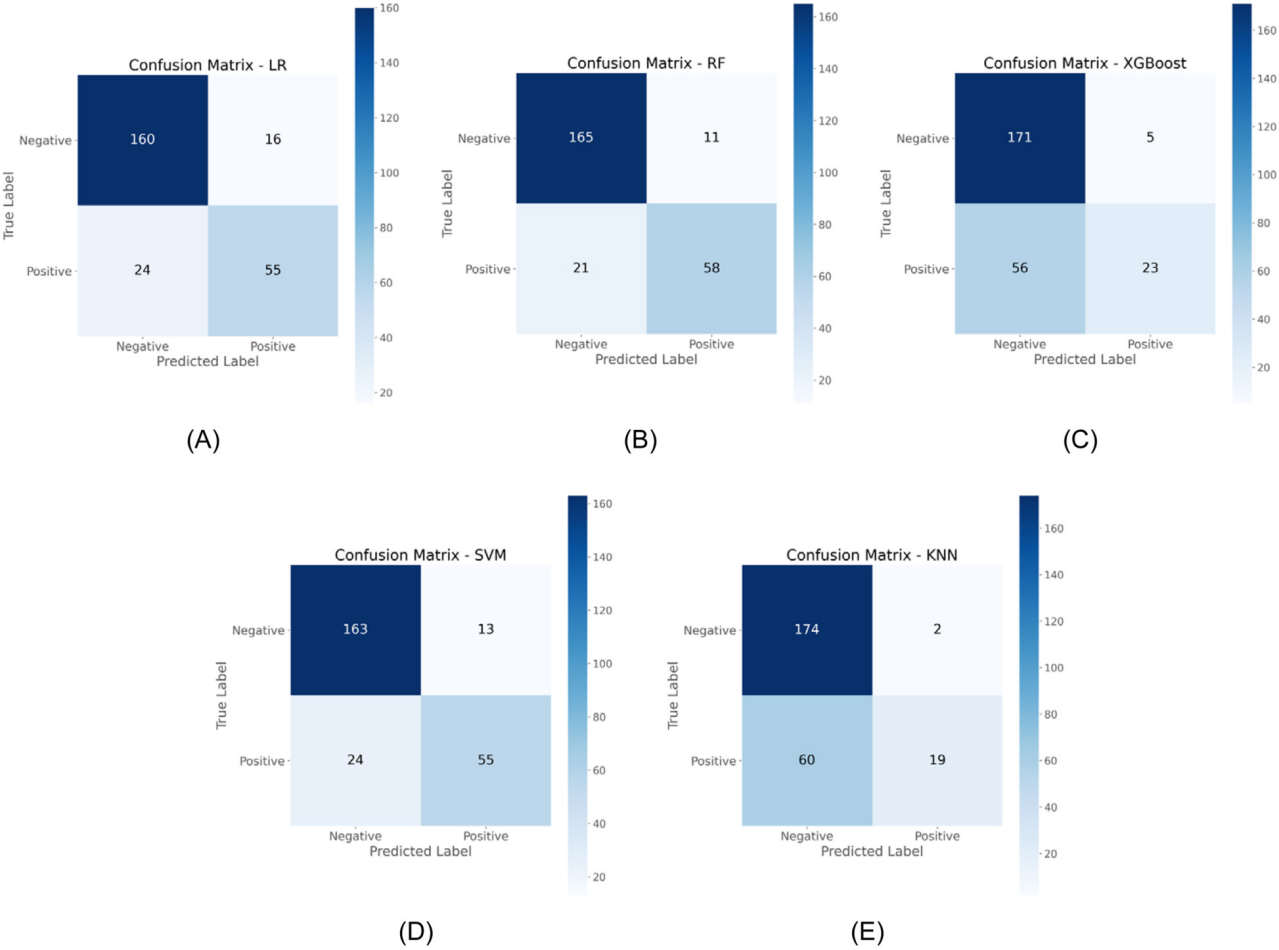
Comparative confusion matrices displaying classification performance of five ML algorithms in the testing cohort. **(A)** LR; **(B)** RF; **(C)** XGBoost; **(D)** SVM; **(E)** KNN.

Confusion matrix analysis further validated these findings, as demonstrated in Figure 3, with RF correctly identifying 58 OME cases while misclassifying only 21 OME patients as non-OME, in contrast to SVM's performance of 55 correct and 24 misclassified OME cases. Additionally, RF demonstrated enhanced specificity, accurately classifying 165 non-OME cases with merely 11 false positives, surpassing SVM's 163 correct non-OME classifications with 13 false positives. As summarized in Table 2, RF achieved superior performance metrics across critical parameters, including sensitivity (0.734), specificity (0.938), accuracy (0.875), and Cohen's kappa coefficient (0.696). Through this comprehensive evaluation process, RF emerged as the optimal algorithm for OME risk stratification in pediatric patients with AH.

**Table 2:** Performance metrics of ML models in the testing cohort.

Model	Sensitivity	Specificity	PPV	NPV	Accuracy	Kappa
LR	0.696 (0.621–0.765)	0.909 (0.870–0.940)	0.775 (0.702–0.839)	0.870 (0.831–0.903)	0.843 (0.803–0.878)	0.623 (0.557–0.689)
SVM	0.696 (0.613–0.762)	0.926 (0.891–0.953)	0.809 (0.738–0.869)	0.872 (0.833–0.905)	0.855 (0.816–0.888)	0.647 (0.582–0.712)
RF	0.734 (0.662–0.798)	0.938 (0.905–0.963)	0.841 (0.775–0.896)	0.887 (0.850–0.918)	0.875 (0.838–0.906)	0.696 (0.634–0.758)
XGBoost	0.291 (0.230–0.360)	0.972 (0.946–0.988)	0.821 (0.715–0.900)	0.753 (0.708–0.795)	0.761 (0.716–0.803)	0.320 (0.241–0.399)
KNN	0.241 (0.185–0.305)	0.989 (0.970–0.997)	0.905 (0.780–0.972)	0.744 (0.699–0.786)	0.757 (0.712–0.799)	0.287 (0.211–0.363)

Values are shown as estimates with 95% confidence intervals. ML, machine learning; PPV, positive predictive value; NPV, negative predictive value; LR, logistic regression; SVM, support vector machine; RF, random forest; XGBoost, eXtreme Gradient Boosting; KNN, K-nearest neighbors.

### Model interpretability and ablation analysis

**Figure 4:**
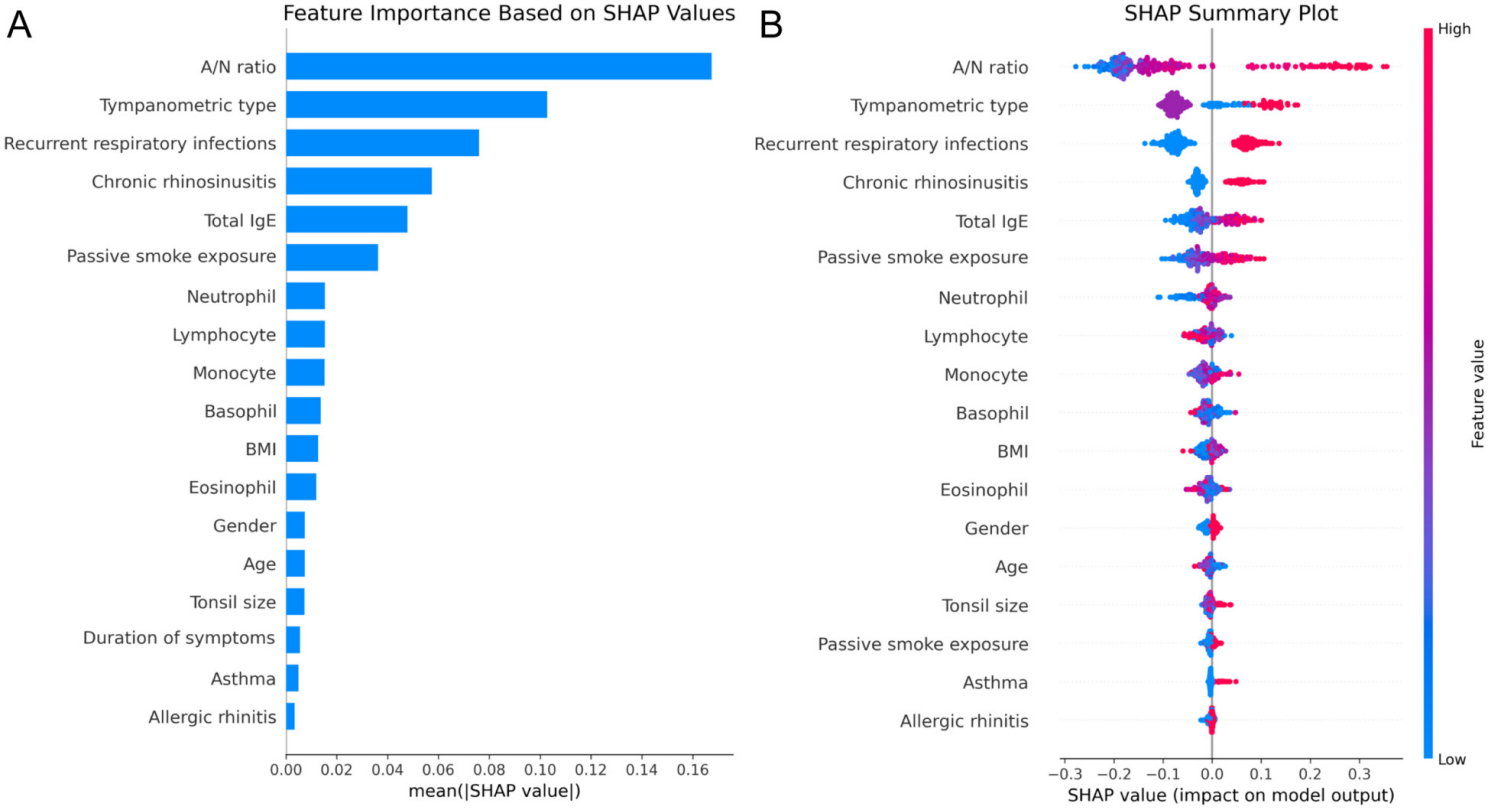
SHAP analysis of the RF model. **(A)** Feature importance ranking based on mean absolute SHAP values. **(B)** SHAP summary plot illustrating feature effects on model output.

SHAP analysis of the optimal RF model revealed the relative influence of predictor variables on OME risk assessment. Feature importance quantification (Figure 4A) identified A/N ratio as the predominant predictor with substantially higher impact than other variables, followed by tympanometric type and history of recurrent respiratory infections. The top five factors also included chronic rhinosinusitis and total IgE levels, reflecting the multifactorial etiology of OME in AH children. The SHAP summary plot (Figure 4B) demonstrated that elevated A/N ratio strongly predicted OME occurrence, while abnormal tympanometric findings (particularly Type B) exhibited consistent association with positive classification.

Ablation experiments validated these contribution patterns, with sequential elimination of features producing systematic performance degradation proportional to SHAP-derived importance rankings (Table 3). Removal of A/N ratio caused the most substantial decline in sensitivity (from 0.734 to 0.519) while preserving specificity, indicating its critical role in identifying true OME cases. Similarly, eliminating tympanometric type reduced sensitivity to 0.544, underscoring its complementary diagnostic value. These findings demonstrate that structural factors and functional measurements provide the foundation for accurate OME prediction, while inflammatory and immunological parameters contribute additional discriminative value through interactions with primary predictors.

**Table 3:** Ablation analysis results showing performance metrics with sequential feature removal from the RF model.

Ablated feature	Sensitivity	Specificity	PPV	NPV	Accuracy	Kappa
None (Baseline)	0.734 (0.662–0.798)	0.938 (0.905–0.963)	0.841 (0.775–0.896)	0.887 (0.850–0.918)	0.875 (0.838–0.906)	0.696 (0.634–0.758)
A/N ratio	0.519 (0.447–0.590)	0.938 (0.901–0.954)	0.788 (0.710–0.854)	0.813 (0.770–0.851)	0.808 (0.767–0.845)	0.504 (0.435–0.573)
Tympanometric type	0.544 (0.472–0.615)	0.943 (0.911–0.967)	0.811 (0.737–0.872)	0.822 (0.780–0.859)	0.820 (0.780–0.855)	0.536 (0.468–0.604)
Recurrent respiratory infections	0.570 (0.498–0.640)	0.949 (0.918–0.971)	0.833 (0.763–0.890)	0.831 (0.790–0.867)	0.831 (0.792–0.866)	0.568 (0.501–0.635)
Chronic rhinosinusitis	0.709 (0.637–0.774)	0.943 (0.911–0.967)	0.848 (0.782–0.902)	0.878 (0.840–0.910)	0.871 (0.833–0.903)	0.683 (0.620–0.746)
Total IgE	0.709 (0.624–0.767)	0.938 (0.905–0.963)	0.836 (0.769–0.891)	0.878 (0.840–0.910)	0.867 (0.829–0.900)	0.675 (0.612–0.738)
Passive smoke exposure	0.696 (0.624–0.762)	0.949 (0.918–0.971)	0.859 (0.795–0.910)	0.874 (0.836–0.906)	0.871 (0.830–0.907)	0.681 (0.618–0.744)

Values are shown as estimates with 95% confidence intervals. PPV, positive predictive value; A/N ratio, adenoid-to-nasopharyngeal ratio; IgE, immunoglobulin E.

## Discussion

The diagnosis of OME in AH children remains challenging due to limitations in existing screening modalities, particularly conventional tympanometry which exhibits variable diagnostic performance. In this study, a ML-based diagnostic tool was developed and validated that integrates clinical, demographic, and acoustic immittance parameters to enhance identification of OME in AH children. The optimal RF model demonstrated excellent discrimination, robust inter-rater agreement, and superior clinical utility in the testing cohort. Unlike previous studies that predominantly utilized LR for linear predictions, this investigation conducted a comprehensive comparison of multiple ML algorithms, enabling thorough examination of both linear and non-linear associations between variables and clinical outcomes. This model offers clinicians an interpretable diagnostic instrument that effectively categorizes patients according to anatomical, functional, and inflammatory parameters without requiring specialized equipment or invasive procedures.

The diagnosis and management of OME in pediatric populations constitute significant public health concerns with substantial developmental consequences. Chronic OME, commonly observed in children with AH, represents a primary cause of acquired hearing impairment during critical developmental periods (32). This auditory deficit often leads to developmental complications including speech delay, communication difficulties, behavioral abnormalities, and educational challenges (33, 34). Untreated persistent OME may evolve to more severe middle ear conditions, including adhesive otitis media, tympanosclerosis, cholesterol granuloma, and acquired primary cholesteatoma. OME identification in pediatric AH patients presents considerable diagnostic difficulties, as young children typically cannot adequately express subtle auditory deficits due to limited metacognitive awareness and language capabilities (35). Parental detection of hearing impairment often occurs only after significant auditory deterioration or when attention deficits become evident in educational environments (36). Tympanometric assessment, although widely used as a non-invasive screening method, exhibits notable diagnostic limitations (10, 37), demonstrated by current findings wherein 11.8% of confirmed OME cases presented with Type A tympanograms (conventionally interpreted as normal), while 13.0% of children without OME displayed Type B tympanograms (traditionally associated with middle ear effusion). These diagnostic inconsistencies highlight the need for improved diagnostic methodologies that overcome the limitations of single-parameter assessment approaches.

In this investigation, the comparative analysis of five ML algorithms identified RF as the optimal approach for OME diagnosis in AH children, demonstrating superior performance across multiple evaluation metrics. The RF model achieved excellent discrimination, balanced calibration, and superior clinical utility, significantly outperforming conventional LR approaches (Figure 2). While LR exhibited acceptable diagnostic accuracy, its inferior PPV highlighted the limitations of linear modeling in capturing complex pathophysiological relationships. To our knowledge, this study represents the first AI-based predictive model for OME risk stratification in AH children. While previous studies have identified individual risk factors using conventional statistical methods (17, 38, 39), our RF model integrates multiple variables to achieve superior predictive performance.

SHAP analysis provided crucial insights into the multifactorial etiology of OME in this population, identifying A/N ratio and tympanometric type as predominant diagnostic indicators. This aligns with recent studies, which identified adenoid grade as a primary risk factor (16, 17). The substantial contribution of these parameters supports established pathophysiological mechanisms whereby AH mechanically obstructs the Eustachian tube orifice, creating negative middle ear pressure with subsequent effusion formation (40, 41). Additionally, inflammatory parameters including recurrent respiratory infections and chronic rhinosinusitis emerged as significant contributors to diagnostic accuracy, consistent with findings from Restuti et al. (38) and previous investigations confirming that bacterial colonization may exert greater influence on OME development than mechanical obstruction alone, with inflammatory responses disrupting ciliary transport and Eustachian tube function (42–44). Immunological parameters (Total IgE) and environmental exposures (passive smoke) demonstrated modest but clinically relevant contributions to the diagnostic model, consistent with prior research indicating tobacco smoke exposure adversely affects both innate and adaptive immunity in children while directly impairing mucociliary clearance through increased goblet cell proliferation and mucus production (45, 46). Importantly, our integrated ML approach enables simultaneous consideration of multiple interacting variables, providing superior predictive performance compared to traditional single-factor analyses while maintaining clinical interpretability through SHAP methodology.

The developed ML-based diagnostic framework provides clinicians with a practical decision support tool that integrates routinely collected clinical parameters to improve OME identification in children with AH. In clinical practice, physicians can implement this model by entering standard patient data to generate an OME probability score that enables risk stratification, allowing clinicians to identify high-risk children who would benefit from immediate confirmatory testing vs. low-risk cases suitable for routine monitoring. For busy clinical practices, this tool offers particular value in optimizing resource allocation by identifying which patients require immediate specialist consultation vs. those who can be safely monitored with standard care protocols. The model can be easily implemented through simple electronic interfaces without requiring additional equipment or specialized training, making it accessible across diverse healthcare settings from community clinics to academic medical centers. Furthermore, the transparent nature of the model allows physicians to understand which specific factors contribute most to OME risk in individual patients, facilitating more informed discussions with families about diagnostic recommendations and treatment plans while improving overall diagnostic confidence in this challenging pediatric population.

Despite the promising results, several methodological limitations warrant consideration. The retrospective single-center design imposed constraints on both generalizability and variable selection. The analysis was restricted to routinely collected clinical parameters, necessarily excluding potentially influential socioeconomic and environmental determinants such as breastfeeding duration and household allergen exposures. Similarly, we were unable to incorporate potentially significant biochemical markers, particularly vitamin D3 levels, despite emerging evidence supporting their association with OME pathogenesis (47, 48). Furthermore, while otoscopic examination represents a routine clinical assessment, these findings were not incorporated due to the challenges in pediatric patient cooperation and the inherent subjectivity in interpretation across clinicians. Additionally, although our internal validation demonstrated robust predictive metrics, the model lacks external validation across diverse clinical settings, geographic regions, and patient populations. This validation gap limits immediate broad clinical implementation. Future research directions should address these limitations through prospective multicenter validation studies, incorporation of comprehensive variable panels including standardized otoscopic findings, socioeconomic factors, and relevant biomarkers, and evaluation of model performance across seasonal variations and diverse clinical contexts. Such refinements would strengthen diagnostic precision and facilitate practical implementation of machine learning approaches in routine pediatric otolaryngology practice.

In conclusion, an RF-based diagnostic model was established that effectively identifies OME in children with AH through integration of accessible clinical parameters. Comparative analysis revealed RF superiority in capturing complex pathophysiological relationships between variables and clinical outcomes. This model addresses limitations in conventional tympanometric assessment by reducing misclassifications, thereby providing a reliable diagnostic instrument for clinical implementation. The framework offers significant value in pediatric populations where symptom reporting remains challenging and early detection prevents developmental sequelae. Through enhanced diagnostic confidence and improved clinical decision-making, this approach advances evidence-based management of OME in the pediatric AH population.

## Data Availability

The original contributions presented in the study are included in the article/Supplementary Material, further inquiries can be directed to the corresponding author.
