# Supplementary material for: Comparative evaluation of machine learning models for enhancing diagnostic accuracy of otitis media with effusion in children with adenoid hypertrophy
Source: Front Pediatr. 2025 Jun 19;13:1614495. doi: 10.3389/fped.2025.1614495 (PMC12222205; doi:10.3389/fped.2025.1614495)
Supplement: Supplementary file 1 [file Datasheet1.pdf]

Supplementary

Table S1. Comparison of demographic and clinical characteristics between training and testing cohorts.

Variable		Training cohort (n=592)	Testing cohort (n=255)	P-value
Group, n(%)	AH	409 (69.1%)	176 (69.0%)	1.000[b]
	AH+OME	183 (30.9%)	79 (31.0%)	
Age, years		5 (3.0, 6.0)	5 (4, 6)	0.124[a]
BMI, kg/m^2		16.7 (14.6, 18.5)	16.6 (14.1, 18.5)	0.356[a]
Duration of symptoms, months		15 (9, 22)	16 (10, 23)	0.246[a]
Gender, n(%)	Female	274 (46.3%)	118 (46.3%)	1.000[b]
	Male	318 (53.7%)	137 (53.7%)	
Tonsil size, n(%)	Grade 0	47 (7.9%)	21 (8.2%)	0.655[b]
	Grade 1	114 (19.3%)	49 (19.2%)	
	Grade 2	229 (38.7%)	99 (38.8%)	
	Grade 3	196 (33.1%)	80 (31.4%)	
	Grade 4	6 (1.0%)	6 (2.4%)	
Allergic rhinitis, n(%)	Yes	416 (70.3%)	175 (68.6%)	0.692[b]
	No	176 (29.7%)	80 (31.4%)	
Asthma, n(%)	Yes	112 (18.9%)	53 (20.8%)	0.593[b]

	No	480 (81.1%)	202 (79.2%)	
Chronic rhinosinusitis, n(%)	Yes	151 (25.5%)	53 (20.8%)	0.165[b]
	No	441 (74.5%)	202 (79.2%)	
Passive smoke exposure, n(%)	Yes	181 (30.6%)	75 (29.4%)	0.798[b]
	No	411 (69.4%)	180 (70.6%)	
Recurrent respiratory infections, n(%)	Yes	423 (71.5%)	170 (66.7%)	0.163[b]
	No	169 (28.5%)	85 (33.3%)	
Basophil, %		0.5 (0.4, 0.7)	0.5 (0.3, 0.7)	0.440[a]
Eosinophil, %		3.3 (2.0, 4.7)	3.3 (2.3, 4.5)	0.750[a]
Lymphocyte, %		45.1 (37.7, 53.0)	45.1 (37.1, 53.4)	0.878[a]
Monocyte, %		4.9 (4.3, 5.7)	5.0 (4.4, 5.7)	0.413[a]
Neutrophil, %		44.3 (38.3, 51.2)	45.8 (39.8, 51.4)	0.174[a]
Total IgE, IU/mL		60.8 (38.1, 83.3)	58.7 (36.7, 88.3)	0.948[a]
A/N ratio		0.82 (0.80, 0.91)	0.8 (0.74, 0.92)	0.518[a]
Tympanometric type, n(%)	Type A	329 (55.6%)	145 (56.9%)	0.321[b]
	Type B	153 (25.8%)	73 (28.6%)	
	Type C	110 (18.6%)	37 (14.5%)	

[a] for Mann-Whitney U test, [b] for chi-square test

Table S2. Optimized hyperparameter configurations for the five ML algorithms.

Model	Parameter	Values
LR	C	0.239
	Penalty	L1
	Solver	saga
	Tolerance	8.183e-5
	max_iter	1409
RF	n_estimators	228
	max_features	None
	max_depth	18
	min_samples_split	13
	min_samples_leaf	3
	min_impurity_decrease	0.0004
SVM	C	0.338
	Kernel	poly
	Gamma	scale
	random_state	42
	tol	0.0005
XGBoost	n_estimators	261
	learning_rate	0.1324
	max_depth	14

	min_child_weight	10
	Gamma	0.9768
	Subsample	0.9934
KNN	algorithm	auto
	metric	manhattan
	n_neighbors	22
	weights	distance

C: Regularization parameter, saga: Stochastic average gradient with acceleration, max_iter: maximum number of iterations, n_estimators: number of trees in ensemble models, min_samples_split = minimum samples required to split an internal node, min_samples_leaf: minimum samples required to be at a leaf node, min_impurity_decrease: threshold for early stopping in tree growth, Gamma: Minimum loss reduction

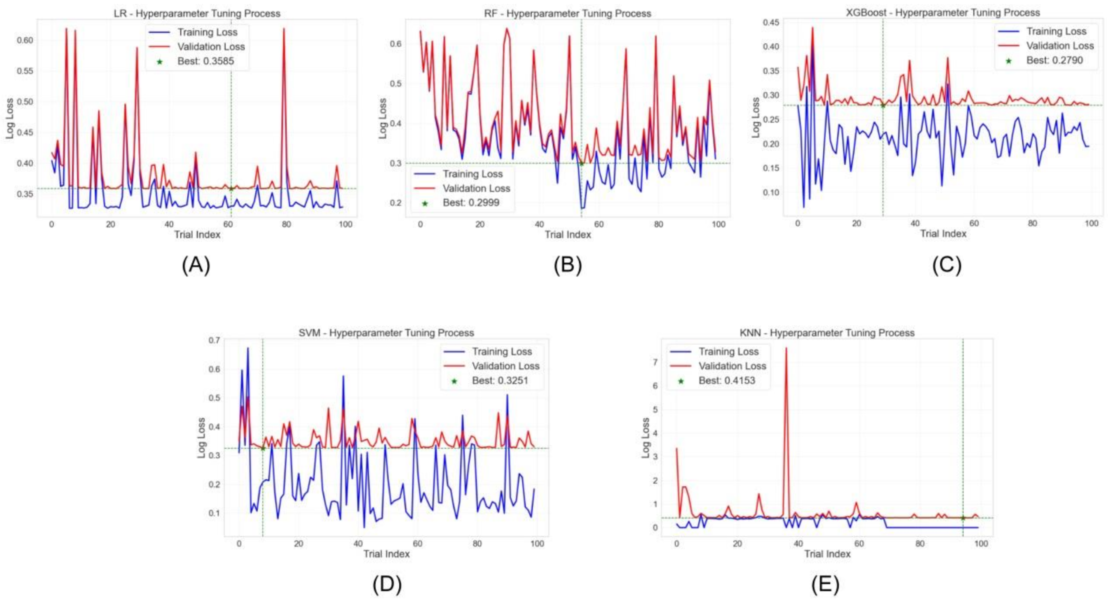

Figure S1. Hyperparameter tuning process for ML models with training and validation loss trajectories.
